# Redetermination of the crystal structure of 3,5-di­methyl­pyrazolium β-octa­molybdate tetra­hydrate

**DOI:** 10.1107/S2056989015022823

**Published:** 2015-12-06

**Authors:** Tatiana R. Amarante, Isabel S. Gonçalves, Filipe A. Almeida Paz

**Affiliations:** a Department of Chemistry, CICECO – Aveiro Institute of Materials, University of Aveiro, 3810-193 Aveiro, Portugal

**Keywords:** crystal structure, 3,5-di­methyl­pyrazolium cations, octa­molybdate(VI) anion, structure redetermination, hydrogen-bonding network

## Abstract

The title compound, (C_5_H_9_N_2_)_4_[Mo_8_O_26_]·4H_2_O, was reported previously from a room-temperature data collection from which only the metal atoms could be refined anisotropically [FitzRoy *et al.* (1989 ▸). *Inorg. Chim. Acta*, **157**, 187–194]. The current redetermination at 180 (2) K models all the non-H atoms with anisotropic displacement parameters and fully describes the supra­molecular N—H⋯O and O—H⋯O hydrogen-bonded network connecting the 3,5-di­methyl­pyrazolium cations, the water mol­ecules of crystallization and the β-octa­molybdate anion. All H atoms involved in the three-dimensional hydrogen-bonding network could be located from difference Fourier maps, with the exception of those of one disordered water mol­ecule, firstly seen in this structural report [refined over two distinct locations with site-occupancy factors of 0.65 (2) and 0.35 (2)]. The complete β-octa­molybdate anion is generated by a crystallographic inversion centre.

## Related literature   

For the previous determination of the title compound at room temperature (Cambridge Structural Database refcode: JAMFEI), see: FitzRoy *et al.* (1989 ▸). For a description of the Cambridge Structural Database, see: Groom & Allen (2014 ▸). For previous studies investigating recovered molybdenum(VI) catalysts, see: Amarante *et al.* (2015 ▸); Lysenko *et al.* (2015 ▸).
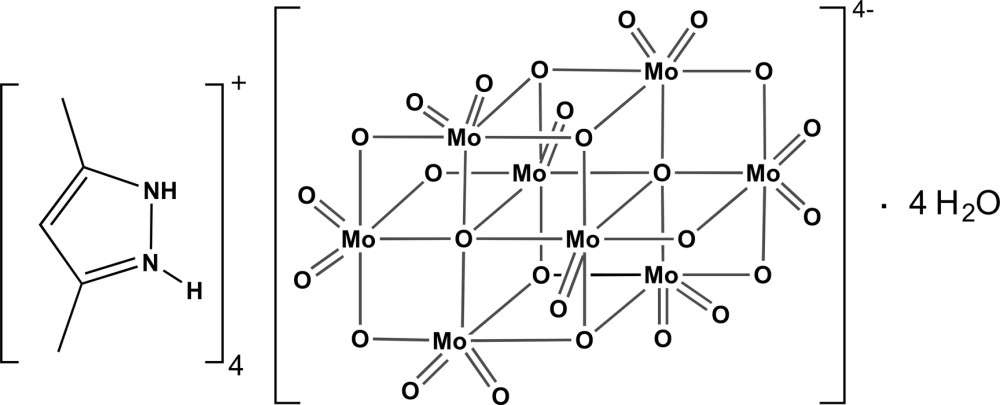


## Experimental   

### Crystal data  

(C_5_H_9_N_2_)_4_[Mo_8_O_26_]·4H_2_O
*M* *_r_* = 1644.15
Triclinic, 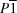

*a* = 10.1105 (9) Å
*b* = 10.7469 (9) Å
*c* = 11.9839 (10) Å
α = 64.103 (3)°
β = 84.272 (3)°
γ = 75.826 (3)°
*V* = 1135.67 (17) Å^3^
*Z* = 1
Mo *K*α radiation
μ = 2.24 mm^−1^
*T* = 180 K
0.20 × 0.14 × 0.01 mm

### Data collection  

Bruker D8 QUEST diffractometer
Absorption correction: multi-scan (*SADABS*; Bruker, 2001 ▸) *T*_min_ = 0.663, *T*_max_ = 0.745
25625 measured reflections
4141 independent reflections
3175 reflections with *I* > 2σ(*I*)
*R*_int_ = 0.042

### Refinement  

*R*[*F* ^2^ > 2σ(*F* ^2^)] = 0.026
*wR*(*F* ^2^) = 0.056
*S* = 1.06
4141 reflections
320 parameters
7 restraints
H atoms treated by a mixture of independent and constrained refinement
Δρ_max_ = 0.70 e Å^−3^
Δρ_min_ = −0.54 e Å^−3^

### 

Data collection: *APEX2* (Bruker, 2012 ▸); cell refinement: *SAINT* (Bruker, 2012 ▸); data reduction: *SAINT*; program(s) used to solve structure: *SHELXS97* (Sheldrick, 2008 ▸); program(s) used to refine structure: *SHELXL2014* (Sheldrick, 2015 ▸); molecular graphics: *DIAMOND* (Brandenburg, 1999 ▸); software used to prepare material for publication: *SHELXL2014*.

## Supplementary Material

Crystal structure: contains datablock(s) I, New_Global_Publ_Block. DOI: 10.1107/S2056989015022823/hb7547sup1.cif

Structure factors: contains datablock(s) I. DOI: 10.1107/S2056989015022823/hb7547Isup2.hkl

**Fig. 1.**
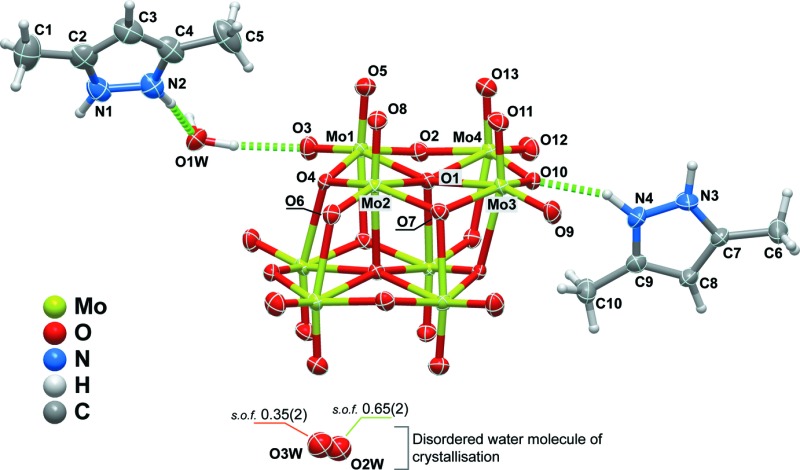
Schematic representation of the molecular entities composing the asymmetric unit of the title compound. The β-octamolybdate anion has been completed by inversion symmetry for the sake of chemical accuracy. All non-hydrogen atoms are represented as displacement ellipsoids drawn at the 60% probability level and hydrogen atoms as small spheres with arbitrary radii. Non-hydrogen atoms belonging to the asymmetric unit have been labelled for clarity. Dashed green broken lines indicate N—H···O and O—H···O hydrogen-bonding interactions (see Table for geometrical details).

. DOI: 10.1107/S2056989015022823/hb7547fig1.tif
Schematic representation of the mol­ecular entities composing the asymmetric unit of the title compound. The β-octa­molybdate anion has been completed by inversion symmetry for the sake of chemical accuracy. All non-hydrogen atoms are represented as displacement ellipsoids drawn at the 60% probability level and hydrogen atoms as small spheres with arbitrary radii. Non-hydrogen atoms belonging to the asymmetric unit have been labelled for clarity. Dashed green broken lines indicate N—H⋯O and O—H⋯O hydrogen-bonding inter­actions (see Table for geometrical details).

**Fig. 2.**
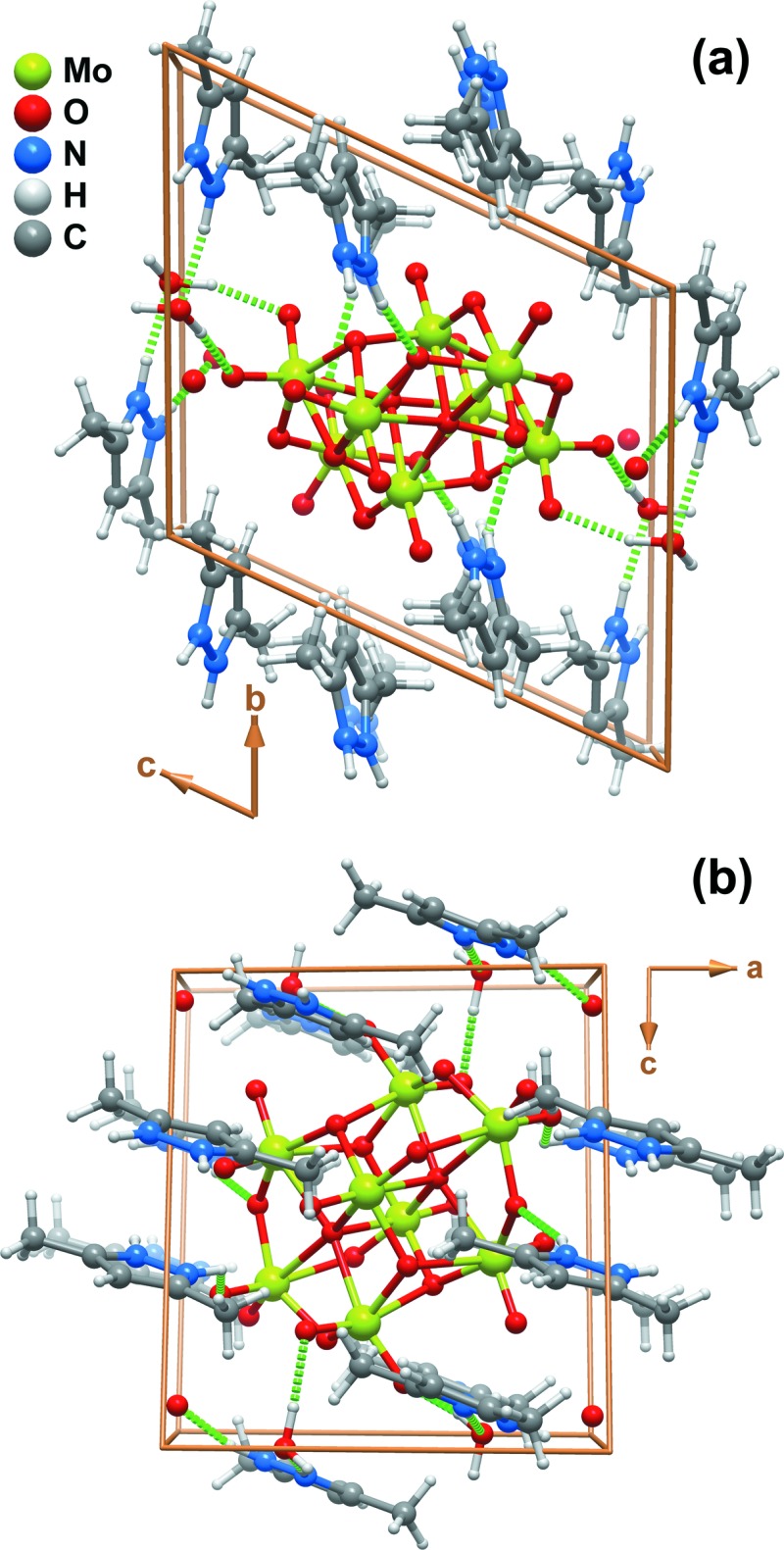
Crystal packing of the title compound viewed in perspective along the (a) [100] (b) [010] directions of the unit cell emphasizing the supramolecular N—H···O and O—H···O hydrogen-bonding interactions (dashed green lines) interconnecting the three types of chemical species present in the crystal structure of the title compound.

(a) (b) . DOI: 10.1107/S2056989015022823/hb7547fig2.tif
Crystal packing of the title compound viewed in perspective along the **(a)** [100] **(b)** [010] directions of the unit cell emphasising the supra­molecular N—H⋯O and O—H⋯O hydrogen-bonding inter­actions (dashed green lines) inter­connecting the three types of chemical species present in the crystal structure of the title compound.

**Fig. 3.**
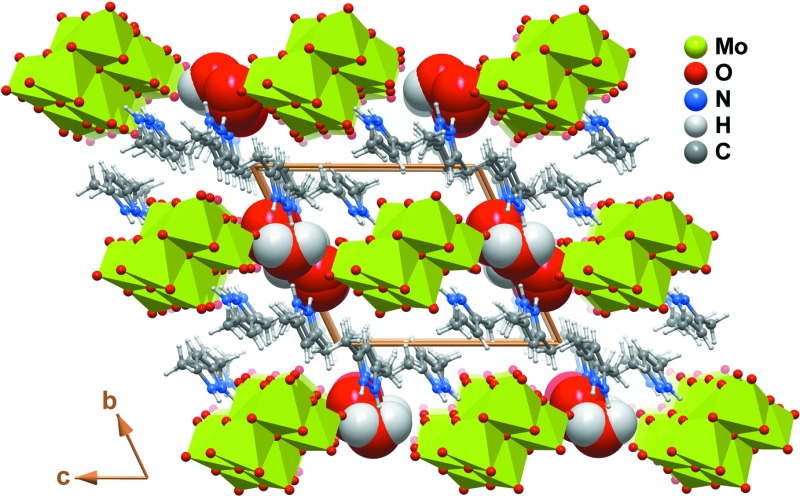
Mixed polyhedral (for the β-octamolybdate anion), ball-and-stick (for the 3,5-dimethylpyrazolium cations) and space filling (for the water molecules of crystallization) schematic representation of the crystal packing of the title compound viewed in perspective along the [100] direction of the unit cell. The Figure illustrates well how the inorganic component of the crystal structure is embedded into an organic matrix, with the entrapped water molecules of crystallization acting as molecular fillers interacting with the hybrid network through hydrogen bonds.

. DOI: 10.1107/S2056989015022823/hb7547fig3.tif
Mixed polyhedral (for the β-octa­molybdate anion), ball-and-stick (for the 3,5-di­methyl­pyrazolium cations) and space filling (for the water mol­ecules of crystallisation) schematic representation of the crystal packing of the title compound viewed in perspective along the [100] direction of the unit cell. The Figure illustrates well how the inorganic component of the crystal structure is embedded into an organic matrix, with the entrapped water mol­ecules of crystallization acting as mol­ecular fillers inter­acting with the hybrid network through hydrogen bonds.

CCDC reference: 1439409

Additional supporting information:  crystallographic information; 3D view; checkCIF report

## Figures and Tables

**Table 1:** Hydrogen-bond geometry (Å, °)

*D*—H⋯*A*	*D*—H	H⋯*A*	*D*⋯*A*	*D*—H⋯*A*
N1—H1⋯O2*W* ^i^	0.94	1.77	2.689 (7)	164
N2—H2⋯O1*W*	0.95	1.84	2.776 (5)	171
N3—H3⋯O13^ii^	0.95	2.28	2.869 (5)	120
N4—H4⋯O10	0.95	1.93	2.801 (4)	152
O1*W*—H1*X*⋯O5^iii^	0.95	1.88	2.785 (4)	160
O1*W*—H1*Y*⋯O3	0.94	1.91	2.848 (4)	172

Symmetry codes: (i) 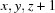; (ii) 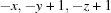; (iii) 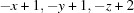.

## supplementary crystallographic information

### S1. Context and introduction

Research efforts in the development and application of new hybrid molybdenum(vi) heterogeneous catalysts require on a daily basis the recovery of used materials and their characterization in the solid state to check for structural modifications of the employed compound (Amarante *et al.*, 2015; Lysenko *et al.*, 2015). Often, the use of drastic experimental conditions leads to the formation of secondary products, which crystallize in the medium as trace amounts of impurity compounds. It is, thus, imperative that most of these possible products are fully described in the solid state in the most accurate fashion.

The title compound, (C_5_H_9_N_2_)_4_[Mo_8_O_26_]^.^4(H_2_O), was previously reported by FitzRoy *et al.* (1989). Besides the fact that the authors did not fully elucidate the hydrogen bonding network of this material (hydrogen atoms were placed geometrically) and that only the metallic centers could be refined anisotropically (from a room-temperature determination), the unit-cell parameters reported in the main text and in the abstract do not match (*viz.* the *c* axis length). A search and match at the Cambridge Structural Database (Groom *et al.*, 2014) also seems to ignore the presence of Mo(VI) metal centers. In this context, we decided to recollect the crystal structure of the title compound at low temperature to fully elucidate its finer structural details.

### S2. Structure description

The asymmetric unit of the title compound is composed of two 3,5-di­methyl­pyrazolium cations, (C_5_H_9_N_2_)^+^, one half of the β-o­cta­molybdate anion, β-[Mo_8_O_26_]^4−^, and two water molecules of crystallization. Noteworthy, while one water molecule was fully located in its crystallographic position and even the associated hydrogen atoms found from difference Fourier maps, the other was found to be disordered over two distinct locations, O2W and O3W (Fig. 1). This feature was not disclosed in the previous structural determination by FitzRoy *et al.* (1989).

The molecular geometrical parameters for the β-o­cta­molybdate anion are typical, exhibiting the usual four families of Mo—O bonds: Mo—O*t* to terminal oxido groups [bond distances in the 1.691 (3)–1.715 (3) Å range]; Mo—O*b* to µ_2_-bridging oxido groups [bond distances in the 1.754 (3)–2.268 (3) Å range]; Mo—O*c* to µ_3_-bridging oxido groups [bond distances in the 1.953 (3)–2.351 (3) Å range]; Mo—O*c* to µ_5_-bridging oxido groups [bond distances in the 2.146 (3)–2.435 (3) Å range]. The four crystallographically independent Mo(VI) metal centers are thus hexacoordinated in a typical {MoO_6_} fashion resembling highly distorted o­cta­hedra: while this *trans* inter­nal O—Mo—O o­cta­hedral angles were found in the 145.29 (11)–173.17 (12)° range, the *cis* angles refined instead in the 70.01 (9)–105.85 (14)° inter­val. We note that this wide dispersion for the inter­nal o­cta­hedral angles is a notable and well known consequence of the marked *trans* effect created by the terminal oxido groups which displace the metal centers from the center of the o­cta­hedra.

The β-o­cta­molybdate anion, located in the center of the unit cell, inter­acts with the remaining chemical species through a series of both electrostatic inter­actions and hydrogen bonds (Fig. 2). One crystallographically independent 3,5-di­methyl­pyrazolium cation donates both the hydrogen atoms bound to nitro­gen to form two strong [D···A distances of 2.801 (4) and 2.869 (5) Å] and relatively directional [<(DHA) angles of 120 and 152°] charged N—H···O inter­actions with the polyoxoanion. The other cation has, however, a completely distinct behaviour: the same hydrogen atoms are instead donated in similar (strong and highly directional) inter­actions with neighbouring water molecules: while the D···A distances are 2.689 (7) and 2.776 (5) Å, the <(DHA) inter­action angles are close to linearity, being 171 and 164°. The water molecules are instead inter­acting with the β-o­cta­molybdate anion as depicted in both Figs. 1 and 2. Indeed, as depicted in Fig. 3 the water molecules play a decisive role in the overall crystal packing, acting as molecular fillers to effectively occupy the available space left from the arrangement of inorganic anions and organic cations.

### S3. Synthesis and crystallization

All chemicals were purchased from commercial sources and used as received without additional purification steps.

A Teflon-lined stainless steel vessel was charged with a reaction mixture composed of MoO_3_ (0.34 g, 2.43 mmol), 3,5-di­methyl­pyrazole (0.11 g, 1.21 mmol) and water (**ca** 25 ml) and heated in an oven at 160 °C for 26 h. The resultant blueish solid was filtered from the aqueous mother liquor and washed with an excess of water and (4×10 ml) di­ethyl ether, dried at ambient temperature and characterized in the solid state. Colourless plates of the title compound were directly harvested from the walls of the Teflon vessel.

Selected FT—IR (KBr, cm^−1^): ñ = 948 (*vs*), 910 (*vs*), 843 (*s*), 733 (*s*), 714 (*s*), 663 (*s*).

### S4. Refinement details

Hydrogen atoms bound to carbon atoms were placed at idealized positions with C—H = 0.95 or 0.98 Å (for the aromatic and methyl groups, respectively), and included in the final structural model in riding-motion approximation with the isotropic thermal displacement parameters fixed at 1.2 or 1.5×*U*_eq_, respectively, of the carbon atom to which they are attached.

Hydrogen atoms associated with nitro­gen atoms have been directly located from difference Fourier maps and were included in the model with the N—H distances restrained to 0.95 (1) Å in order to ensure a chemically reasonable environment for these moieties. These hydrogen atoms were modelled with the isotropic thermal displacement parameters fixed at 1.5×*U*_eq_(N).

A total of two water molecules of crystallization were directly located from difference Fourier maps. Though O1W was included in the final structural model by assuming full site occupancy and a typical anisotropic displacement behaviour, the second molecule was found to be disordered over two close crystallographic positions: O2W and O3W. These species were included in the structural model with linked site occupancy [which ultimately refined to 0.65 (2) and 0.35 (2), respectively] and by assuming an independent isotropic displacement behaviour. For O1W the two hydrogen atoms were markedly visible in difference Fourier maps and were included in the final model with the O—H and H···H distances restrained to 0.95 (1) and 1.55 (1) Å, respectively, in order to ensure a chemically reasonable geometry for this molecule. These hydrogen atoms were modelled with the isotropic thermal displacement parameters fixed at 1.5×*U*_eq_(O1W).

### Crystal data

**Table:**

(C_5_H_9_N_2_)_4_[Mo_8_O_26_]·4H_2_O	*Z* = 1
*M**_r_* = 1644.15	*F*(000) = 796
Triclinic, *P*1	*D*_x_ = 2.404 Mg m^−^^3^
*a* = 10.1105 (9) Å	Mo *K*α radiation, λ = 0.71073 Å
*b* = 10.7469 (9) Å	Cell parameters from 9938 reflections
*c* = 11.9839 (10) Å	θ = 2.6–25.4°
α = 64.103 (3)°	µ = 2.24 mm^−^^1^
β = 84.272 (3)°	*T* = 180 K
γ = 75.826 (3)°	Plate, colourless
*V* = 1135.67 (17) Å^3^	0.20 × 0.14 × 0.01 mm

### Data collection

**Table:**

Bruker D8 QUEST diffractometer	4141 independent reflections
Radiation source: Sealed tube	3175 reflections with *I* > 2σ(*I*)
Multi-layer X-ray mirror monochromator	*R*_int_ = 0.042
Detector resolution: 10.4167 pixels mm^-1^	θ_max_ = 25.4°, θ_min_ = 3.7°
ω / φ scans	*h* = −12→12
Absorption correction: multi-scan (*SADABS*; Bruker, 2001)	*k* = −12→12
*T*_min_ = 0.663, *T*_max_ = 0.745	*l* = −14→14
25625 measured reflections	

### Refinement

**Table:**

Refinement on *F*^2^	7 restraints
Least-squares matrix: full	Hydrogen site location: mixed
*R*[*F*^2^ > 2σ(*F*^2^)] = 0.026	H atoms treated by a mixture of independent and constrained refinement
*wR*(*F*^2^) = 0.056	*w* = 1/[σ^2^(*F*_o_^2^) + (0.0188*P*)^2^ + 2.2481*P*] where *P* = (*F*_o_^2^ + 2*F*_c_^2^)/3
*S* = 1.06	(Δ/σ)_max_ = 0.001
4141 reflections	Δρ_max_ = 0.70 e Å^−^^3^
320 parameters	Δρ_min_ = −0.54 e Å^−^^3^

### Special details

**Table:**

Geometry. All e.s.d.'s (except the e.s.d. in the dihedral angle between two l.s. planes) are estimated using the full covariance matrix. The cell e.s.d.'s are taken into account individually in the estimation of e.s.d.'s in distances, angles and torsion angles; correlations between e.s.d.'s in cell parameters are only used when they are defined by crystal symmetry. An approximate (isotropic) treatment of cell e.s.d.'s is used for estimating e.s.d.'s involving l.s. planes.

### Fractional atomic coordinates and isotropic or equivalent isotropic displacement parameters (Å^2^)

**Table:**

	*x*	*y*	*z*	*U*_iso_*/*U*_eq_	Occ. (<1)
Mo1	0.44983 (3)	0.44979 (4)	0.75253 (3)	0.01430 (9)	
Mo2	0.45658 (3)	0.68126 (4)	0.47103 (3)	0.01229 (9)	
Mo3	0.23903 (3)	0.56541 (4)	0.38528 (3)	0.01415 (9)	
Mo4	0.23047 (3)	0.32798 (4)	0.67012 (3)	0.01511 (9)	
O1	0.3753 (3)	0.4944 (3)	0.5589 (2)	0.0142 (6)	
O2	0.3635 (3)	0.3005 (3)	0.7899 (2)	0.0177 (6)	
O3	0.5489 (3)	0.3881 (3)	0.8808 (3)	0.0235 (7)	
O4	0.5539 (3)	0.5839 (3)	0.6298 (2)	0.0145 (6)	
O5	0.3166 (3)	0.5748 (3)	0.7683 (3)	0.0208 (7)	
O6	0.5740 (3)	0.7845 (3)	0.3880 (2)	0.0168 (6)	
O7	0.3838 (3)	0.6770 (3)	0.3281 (2)	0.0153 (6)	
O8	0.3261 (3)	0.7924 (3)	0.5040 (3)	0.0190 (6)	
O9	0.1894 (3)	0.5797 (3)	0.2484 (3)	0.0232 (7)	
O10	0.1936 (3)	0.3896 (3)	0.4970 (2)	0.0160 (6)	
O11	0.1191 (3)	0.6842 (3)	0.4197 (3)	0.0220 (7)	
O12	0.1720 (3)	0.1773 (3)	0.7422 (3)	0.0255 (7)	
O13	0.1090 (3)	0.4568 (3)	0.6928 (3)	0.0213 (7)	
N1	0.7819 (4)	0.8121 (5)	0.9458 (3)	0.0294 (9)	
H1	0.865 (3)	0.747 (4)	0.979 (4)	0.044*	
N2	0.6839 (4)	0.7657 (4)	0.9163 (4)	0.0281 (9)	
H2	0.692 (5)	0.6692 (19)	0.934 (5)	0.042*	
N3	−0.0378 (4)	0.2498 (4)	0.3524 (4)	0.0295 (9)	
H3	−0.116 (3)	0.314 (4)	0.362 (5)	0.044*	
N4	0.0827 (4)	0.2507 (4)	0.3913 (4)	0.0289 (9)	
H4	0.090 (5)	0.314 (4)	0.425 (4)	0.043*	
C1	0.8229 (6)	1.0327 (6)	0.9381 (5)	0.0491 (15)	
H1A	0.8535	0.9870	1.0247	0.074*	
H1B	0.7696	1.1293	0.9183	0.074*	
H1C	0.9023	1.0365	0.8837	0.074*	
C2	0.7364 (5)	0.9494 (5)	0.9191 (4)	0.0319 (11)	
C3	0.6048 (5)	0.9913 (5)	0.8718 (4)	0.0316 (11)	
H3A	0.5471	1.0837	0.8443	0.038*	
C4	0.5743 (5)	0.8733 (5)	0.8726 (4)	0.0312 (11)	
C5	0.4486 (5)	0.8533 (6)	0.8341 (5)	0.0420 (14)	
H5A	0.4728	0.7778	0.8058	0.063*	
H5B	0.4035	0.9420	0.7663	0.063*	
H5C	0.3866	0.8271	0.9047	0.063*	
C6	−0.1363 (5)	0.1101 (5)	0.2791 (5)	0.0329 (12)	
H6A	−0.2222	0.1413	0.3150	0.049*	
H6B	−0.1209	0.0082	0.3021	0.049*	
H6C	−0.1412	0.1624	0.1885	0.049*	
C7	−0.0218 (4)	0.1381 (5)	0.3270 (4)	0.0208 (10)	
C8	0.1130 (4)	0.0663 (5)	0.3502 (4)	0.0225 (10)	
H8	0.1545	−0.0174	0.3399	0.027*	
C9	0.1768 (4)	0.1398 (5)	0.3917 (4)	0.0208 (10)	
C10	0.3202 (4)	0.1135 (5)	0.4290 (5)	0.0303 (11)	
H10A	0.3497	0.2034	0.3936	0.045*	
H10B	0.3787	0.0479	0.3983	0.045*	
H10C	0.3268	0.0717	0.5196	0.045*	
O1W	0.7299 (3)	0.4764 (4)	0.9840 (3)	0.0276 (7)	
H1X	0.711 (5)	0.439 (5)	1.0698 (12)	0.041*	
H1Y	0.676 (4)	0.449 (5)	0.943 (3)	0.041*	
O2W	0.9897 (6)	0.5952 (10)	0.0682 (5)	0.031 (2)*	0.65 (2)
O3W	1.0101 (11)	0.6562 (19)	0.0507 (10)	0.033 (4)*	0.35 (2)

### Atomic displacement parameters (Å^2^)

**Table:**

	*U*^11^	*U*^22^	*U*^33^	*U*^12^	*U*^13^	*U*^23^
Mo1	0.01445 (18)	0.0179 (2)	0.01272 (18)	−0.00473 (15)	0.00061 (14)	−0.00798 (16)
Mo2	0.01153 (18)	0.01158 (19)	0.01458 (18)	−0.00190 (14)	−0.00116 (14)	−0.00643 (15)
Mo3	0.01088 (18)	0.0166 (2)	0.01578 (19)	−0.00309 (15)	−0.00217 (14)	−0.00718 (16)
Mo4	0.01333 (18)	0.0163 (2)	0.01719 (19)	−0.00532 (15)	0.00086 (14)	−0.00759 (16)
O1	0.0124 (14)	0.0157 (15)	0.0150 (14)	−0.0038 (12)	0.0002 (11)	−0.0069 (12)
O2	0.0179 (15)	0.0185 (16)	0.0157 (14)	−0.0065 (12)	0.0021 (12)	−0.0055 (13)
O3	0.0232 (16)	0.0307 (19)	0.0185 (16)	−0.0101 (14)	−0.0013 (13)	−0.0100 (14)
O4	0.0142 (14)	0.0157 (15)	0.0179 (15)	−0.0041 (12)	−0.0013 (11)	−0.0104 (13)
O5	0.0194 (15)	0.0228 (17)	0.0223 (16)	−0.0053 (13)	0.0038 (13)	−0.0122 (14)
O6	0.0184 (15)	0.0146 (15)	0.0171 (15)	−0.0040 (12)	−0.0022 (12)	−0.0058 (13)
O7	0.0128 (14)	0.0170 (16)	0.0150 (14)	−0.0024 (12)	−0.0010 (11)	−0.0060 (12)
O8	0.0186 (15)	0.0155 (16)	0.0227 (16)	−0.0007 (12)	−0.0028 (12)	−0.0091 (13)
O9	0.0201 (16)	0.0316 (19)	0.0194 (16)	−0.0067 (14)	−0.0042 (13)	−0.0110 (14)
O10	0.0146 (14)	0.0169 (16)	0.0199 (15)	−0.0045 (12)	−0.0005 (12)	−0.0101 (13)
O11	0.0159 (15)	0.0214 (17)	0.0273 (17)	−0.0002 (13)	−0.0014 (13)	−0.0110 (14)
O12	0.0243 (16)	0.0219 (17)	0.0313 (18)	−0.0108 (14)	0.0025 (14)	−0.0099 (15)
O13	0.0171 (15)	0.0227 (17)	0.0232 (16)	−0.0038 (13)	0.0018 (13)	−0.0098 (14)
N1	0.023 (2)	0.038 (3)	0.022 (2)	−0.0038 (19)	−0.0028 (17)	−0.0087 (19)
N2	0.027 (2)	0.029 (2)	0.024 (2)	−0.0071 (19)	−0.0013 (17)	−0.0067 (19)
N3	0.020 (2)	0.029 (2)	0.046 (3)	0.0003 (17)	0.0004 (18)	−0.026 (2)
N4	0.026 (2)	0.031 (2)	0.041 (2)	−0.0094 (18)	0.0027 (18)	−0.026 (2)
C1	0.061 (4)	0.053 (4)	0.039 (3)	−0.024 (3)	−0.005 (3)	−0.017 (3)
C2	0.036 (3)	0.035 (3)	0.020 (2)	−0.010 (2)	0.004 (2)	−0.007 (2)
C3	0.032 (3)	0.025 (3)	0.025 (3)	−0.002 (2)	−0.001 (2)	−0.001 (2)
C4	0.027 (3)	0.033 (3)	0.022 (2)	−0.004 (2)	−0.002 (2)	−0.001 (2)
C5	0.028 (3)	0.046 (4)	0.036 (3)	−0.011 (2)	−0.006 (2)	0.000 (3)
C6	0.024 (3)	0.035 (3)	0.050 (3)	−0.003 (2)	−0.008 (2)	−0.027 (3)
C7	0.021 (2)	0.020 (2)	0.026 (2)	−0.0049 (19)	0.0010 (18)	−0.014 (2)
C8	0.023 (2)	0.024 (3)	0.026 (2)	−0.0022 (19)	−0.0022 (19)	−0.017 (2)
C9	0.022 (2)	0.024 (2)	0.019 (2)	−0.0054 (19)	0.0015 (18)	−0.011 (2)
C10	0.025 (2)	0.030 (3)	0.043 (3)	−0.006 (2)	−0.005 (2)	−0.021 (2)
O1W	0.0298 (18)	0.036 (2)	0.0240 (17)	−0.0078 (15)	−0.0003 (14)	−0.0192 (16)

### Geometric parameters (Å, º)

**Table:**

Mo1—O3	1.702 (3)	N2—C4	1.340 (6)
Mo1—O5	1.715 (3)	N2—H2	0.946 (10)
Mo1—O2	1.875 (3)	N3—C7	1.332 (5)
Mo1—O4	1.982 (3)	N3—N4	1.351 (5)
Mo1—O7^i^	2.307 (3)	N3—H3	0.947 (10)
Mo1—O1	2.320 (3)	N4—C9	1.328 (6)
Mo1—Mo2	3.2062 (6)	N4—H4	0.947 (10)
Mo2—O8	1.691 (3)	C1—C2	1.488 (7)
Mo2—O6	1.754 (3)	C1—H1A	0.9800
Mo2—O7	1.953 (3)	C1—H1B	0.9800
Mo2—O4	1.953 (3)	C1—H1C	0.9800
Mo2—O1	2.146 (3)	C2—C3	1.389 (7)
Mo2—O1^i^	2.341 (3)	C3—C4	1.373 (7)
Mo2—Mo3	3.2178 (5)	C3—H3A	0.9500
Mo3—O9	1.695 (3)	C4—C5	1.488 (7)
Mo3—O11	1.695 (3)	C5—H5A	0.9800
Mo3—O10	1.915 (3)	C5—H5B	0.9800
Mo3—O7	1.998 (3)	C5—H5C	0.9800
Mo3—O1	2.341 (3)	C6—C7	1.488 (6)
Mo3—O4^i^	2.351 (3)	C6—H6A	0.9800
Mo4—O12	1.692 (3)	C6—H6B	0.9800
Mo4—O13	1.711 (3)	C6—H6C	0.9800
Mo4—O10	1.928 (3)	C7—C8	1.379 (6)
Mo4—O2	1.943 (3)	C8—C9	1.394 (6)
Mo4—O6^i^	2.268 (3)	C8—H8	0.9500
Mo4—O1	2.435 (3)	C9—C10	1.483 (6)
O1—Mo2^i^	2.341 (3)	C10—H10A	0.9800
O4—Mo3^i^	2.351 (3)	C10—H10B	0.9800
O6—Mo4^i^	2.268 (3)	C10—H10C	0.9800
O7—Mo1^i^	2.307 (3)	O1W—H1X	0.946 (10)
N1—C2	1.332 (6)	O1W—H1Y	0.944 (10)
N1—N2	1.350 (5)	O2W—O3W	0.674 (13)
N1—H1	0.943 (10)		
			
O3—Mo1—O5	104.71 (14)	O12—Mo4—O1	162.52 (12)
O3—Mo1—O2	102.12 (13)	O13—Mo4—O1	91.62 (11)
O5—Mo1—O2	100.72 (13)	O10—Mo4—O1	74.97 (10)
O3—Mo1—O4	99.75 (12)	O2—Mo4—O1	73.62 (10)
O5—Mo1—O4	97.01 (13)	O6^i^—Mo4—O1	70.01 (9)
O2—Mo1—O4	147.18 (11)	Mo2—O1—Mo1	91.68 (9)
O3—Mo1—O7^i^	89.69 (12)	Mo2—O1—Mo2^i^	104.01 (10)
O5—Mo1—O7^i^	163.56 (12)	Mo1—O1—Mo2^i^	97.04 (9)
O2—Mo1—O7^i^	83.57 (11)	Mo2—O1—Mo3	91.54 (10)
O4—Mo1—O7^i^	72.34 (10)	Mo1—O1—Mo3	163.07 (12)
O3—Mo1—O1	161.88 (12)	Mo2^i^—O1—Mo3	98.29 (9)
O5—Mo1—O1	93.03 (11)	Mo2—O1—Mo4	163.99 (13)
O2—Mo1—O1	77.65 (10)	Mo1—O1—Mo4	86.35 (9)
O4—Mo1—O1	74.01 (10)	Mo2^i^—O1—Mo4	91.99 (9)
O7^i^—Mo1—O1	72.23 (9)	Mo3—O1—Mo4	85.98 (8)
O3—Mo1—Mo2	134.82 (10)	Mo1—O2—Mo4	116.94 (14)
O5—Mo1—Mo2	84.85 (10)	Mo2—O4—Mo1	109.13 (12)
O2—Mo1—Mo2	119.63 (8)	Mo2—O4—Mo3^i^	110.39 (11)
O4—Mo1—Mo2	35.14 (7)	Mo1—O4—Mo3^i^	103.30 (11)
O7^i^—Mo1—Mo2	79.31 (7)	Mo2—O6—Mo4^i^	116.79 (14)
O1—Mo1—Mo2	41.99 (7)	Mo2—O7—Mo3	109.07 (13)
O8—Mo2—O6	105.54 (13)	Mo2—O7—Mo1^i^	109.84 (11)
O8—Mo2—O7	101.63 (12)	Mo3—O7—Mo1^i^	104.33 (12)
O6—Mo2—O7	96.27 (12)	Mo3—O10—Mo4	115.92 (13)
O8—Mo2—O4	99.93 (12)	C2—N1—N2	108.8 (4)
O6—Mo2—O4	97.02 (11)	C2—N1—H1	133 (3)
O7—Mo2—O4	150.55 (11)	N2—N1—H1	118 (3)
O8—Mo2—O1	97.27 (12)	C4—N2—N1	108.9 (4)
O6—Mo2—O1	157.19 (11)	C4—N2—H2	128 (3)
O7—Mo2—O1	78.87 (10)	N1—N2—H2	123 (3)
O4—Mo2—O1	78.71 (10)	C7—N3—N4	109.1 (4)
O8—Mo2—O1^i^	173.17 (12)	C7—N3—H3	133 (3)
O6—Mo2—O1^i^	81.21 (11)	N4—N3—H3	117 (3)
O7—Mo2—O1^i^	78.31 (10)	C9—N4—N3	109.5 (4)
O4—Mo2—O1^i^	77.92 (10)	C9—N4—H4	128 (3)
O1—Mo2—O1^i^	75.99 (10)	N3—N4—H4	122 (3)
O8—Mo2—Mo1	88.75 (10)	C2—C1—H1A	109.5
O6—Mo2—Mo1	132.75 (9)	C2—C1—H1B	109.5
O7—Mo2—Mo1	125.17 (8)	H1A—C1—H1B	109.5
O4—Mo2—Mo1	35.73 (8)	C2—C1—H1C	109.5
O1—Mo2—Mo1	46.33 (7)	H1A—C1—H1C	109.5
O1^i^—Mo2—Mo1	85.79 (7)	H1B—C1—H1C	109.5
O8—Mo2—Mo3	89.31 (9)	N1—C2—C3	107.8 (4)
O6—Mo2—Mo3	132.20 (9)	N1—C2—C1	121.8 (5)
O7—Mo2—Mo3	35.93 (8)	C3—C2—C1	130.4 (5)
O4—Mo2—Mo3	125.36 (8)	C4—C3—C2	106.6 (4)
O1—Mo2—Mo3	46.65 (7)	C4—C3—H3A	126.7
O1^i^—Mo2—Mo3	86.76 (6)	C2—C3—H3A	126.7
Mo1—Mo2—Mo3	91.724 (14)	N2—C4—C3	107.8 (4)
O9—Mo3—O11	105.85 (14)	N2—C4—C5	121.2 (5)
O9—Mo3—O10	100.45 (13)	C3—C4—C5	130.9 (5)
O11—Mo3—O10	101.76 (13)	C4—C5—H5A	109.5
O9—Mo3—O7	100.71 (12)	C4—C5—H5B	109.5
O11—Mo3—O7	97.58 (12)	H5A—C5—H5B	109.5
O10—Mo3—O7	146.06 (11)	C4—C5—H5C	109.5
O9—Mo3—O1	159.60 (12)	H5A—C5—H5C	109.5
O11—Mo3—O1	94.36 (12)	H5B—C5—H5C	109.5
O10—Mo3—O1	77.54 (10)	C7—C6—H6A	109.5
O7—Mo3—O1	73.40 (10)	C7—C6—H6B	109.5
O9—Mo3—O4^i^	88.84 (12)	H6A—C6—H6B	109.5
O11—Mo3—O4^i^	163.17 (11)	C7—C6—H6C	109.5
O10—Mo3—O4^i^	83.12 (10)	H6A—C6—H6C	109.5
O7—Mo3—O4^i^	71.11 (10)	H6B—C6—H6C	109.5
O1—Mo3—O4^i^	70.77 (9)	N3—C7—C8	107.4 (4)
O9—Mo3—Mo2	135.69 (10)	N3—C7—C6	121.8 (4)
O11—Mo3—Mo2	85.49 (10)	C8—C7—C6	130.8 (4)
O10—Mo3—Mo2	119.34 (8)	C7—C8—C9	107.0 (4)
O7—Mo3—Mo2	35.00 (7)	C7—C8—H8	126.5
O1—Mo3—Mo2	41.81 (7)	C9—C8—H8	126.5
O4^i^—Mo3—Mo2	78.16 (6)	N4—C9—C8	106.9 (4)
O12—Mo4—O13	105.75 (14)	N4—C9—C10	121.9 (4)
O12—Mo4—O10	103.91 (13)	C8—C9—C10	131.2 (4)
O13—Mo4—O10	97.77 (13)	C9—C10—H10A	109.5
O12—Mo4—O2	101.63 (13)	C9—C10—H10B	109.5
O13—Mo4—O2	97.49 (13)	H10A—C10—H10B	109.5
O10—Mo4—O2	145.29 (11)	C9—C10—H10C	109.5
O12—Mo4—O6^i^	92.59 (12)	H10A—C10—H10C	109.5
O13—Mo4—O6^i^	161.61 (12)	H10B—C10—H10C	109.5
O10—Mo4—O6^i^	78.80 (10)	H1X—O1W—H1Y	109.9 (16)
O2—Mo4—O6^i^	76.95 (11)		
			
O3—Mo1—O2—Mo4	177.15 (15)	N2—N1—C2—C1	−178.3 (4)
O5—Mo1—O2—Mo4	69.40 (17)	N1—C2—C3—C4	0.4 (5)
O4—Mo1—O2—Mo4	−52.1 (3)	C1—C2—C3—C4	179.0 (5)
O7^i^—Mo1—O2—Mo4	−94.55 (15)	N1—N2—C4—C3	1.4 (5)
O1—Mo1—O2—Mo4	−21.39 (13)	N1—N2—C4—C5	−179.1 (4)
Mo2—Mo1—O2—Mo4	−20.75 (18)	C2—C3—C4—N2	−1.1 (5)
O8—Mo2—O6—Mo4^i^	178.38 (13)	C2—C3—C4—C5	179.4 (5)
O7—Mo2—O6—Mo4^i^	−77.67 (14)	N4—N3—C7—C8	−0.1 (5)
O4—Mo2—O6—Mo4^i^	75.99 (14)	N4—N3—C7—C6	−178.6 (4)
O1—Mo2—O6—Mo4^i^	−1.5 (4)	N3—C7—C8—C9	0.4 (5)
O1^i^—Mo2—O6—Mo4^i^	−0.57 (12)	C6—C7—C8—C9	178.7 (5)
Mo1—Mo2—O6—Mo4^i^	75.31 (16)	N3—N4—C9—C8	0.5 (5)
Mo3—Mo2—O6—Mo4^i^	−78.01 (15)	N3—N4—C9—C10	179.3 (4)
C2—N1—N2—C4	−1.1 (5)	C7—C8—C9—N4	−0.6 (5)
C7—N3—N4—C9	−0.2 (5)	C7—C8—C9—C10	−179.2 (5)
N2—N1—C2—C3	0.4 (5)		

Symmetry code: (i) −*x*+1, −*y*+1, −*z*+1.

### Hydrogen-bond geometry (Å, º)

**Table:**

*D*—H···*A*	*D*—H	H···*A*	*D*···*A*	*D*—H···*A*
N1—H1···O2*W*^ii^	0.94	1.77	2.689 (7)	164
N2—H2···O1*W*	0.95	1.84	2.776 (5)	171
N3—H3···O13^iii^	0.95	2.28	2.869 (5)	120
N4—H4···O10	0.95	1.93	2.801 (4)	152
O1*W*—H1*X*···O5^iv^	0.95	1.88	2.785 (4)	160
O1*W*—H1*Y*···O3	0.94	1.91	2.848 (4)	172

Symmetry codes: (ii) *x*, *y*, *z*+1; (iii) −*x*, −*y*+1, −*z*+1; (iv) −*x*+1, −*y*+1, −*z*+2.
