# Supplementary figures and images for: Redetermination of the crystal structure of 3,5-di­methyl­pyrazolium β-octa­molybdate tetra­hydrate
Source: Acta Crystallogr E Crystallogr Commun. 2015 Dec 6;71(Pt 12):m244–5. doi: 10.1107/S2056989015022823 (PMC4719855; doi:10.1107/S2056989015022823)

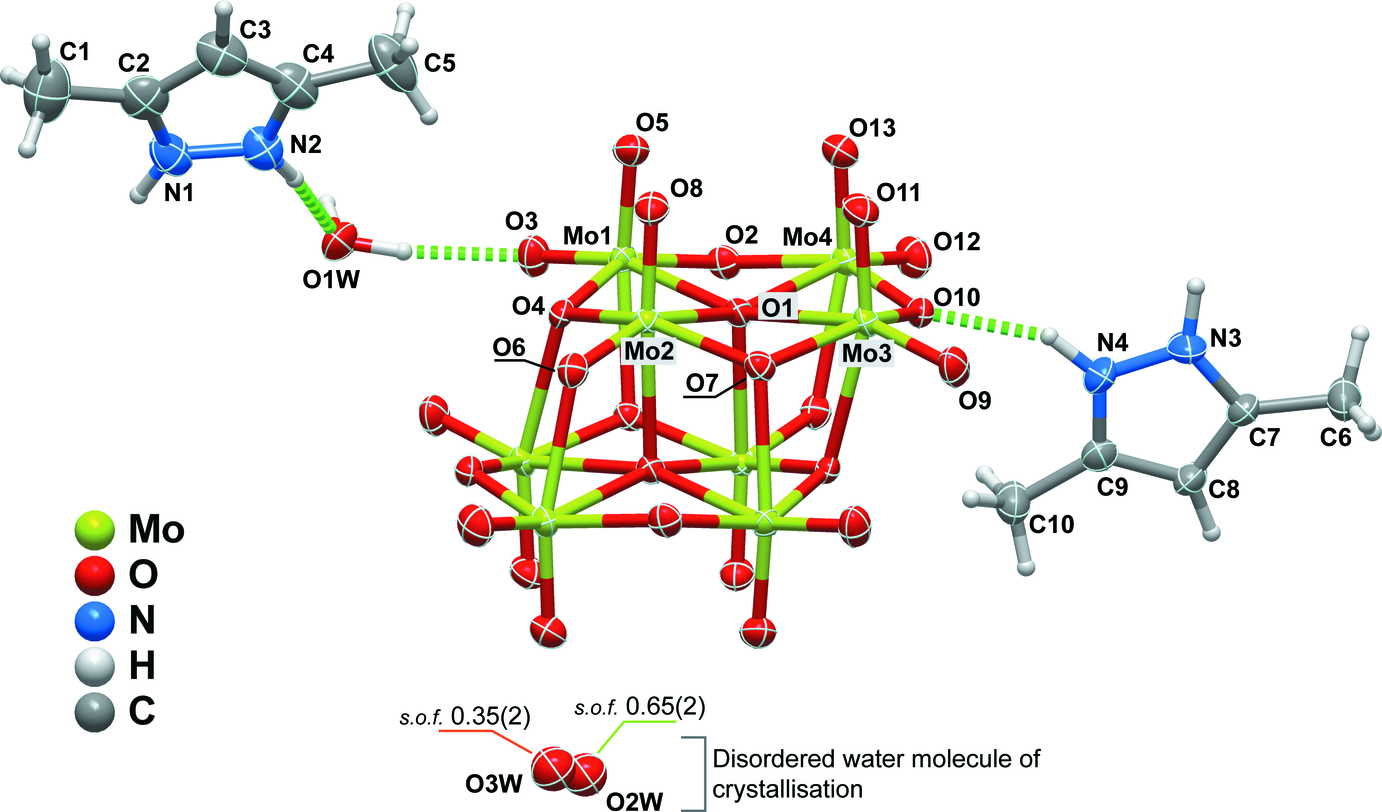

Supplement: Supplementary file 3 [file e-71-0m244-fig1.tif]

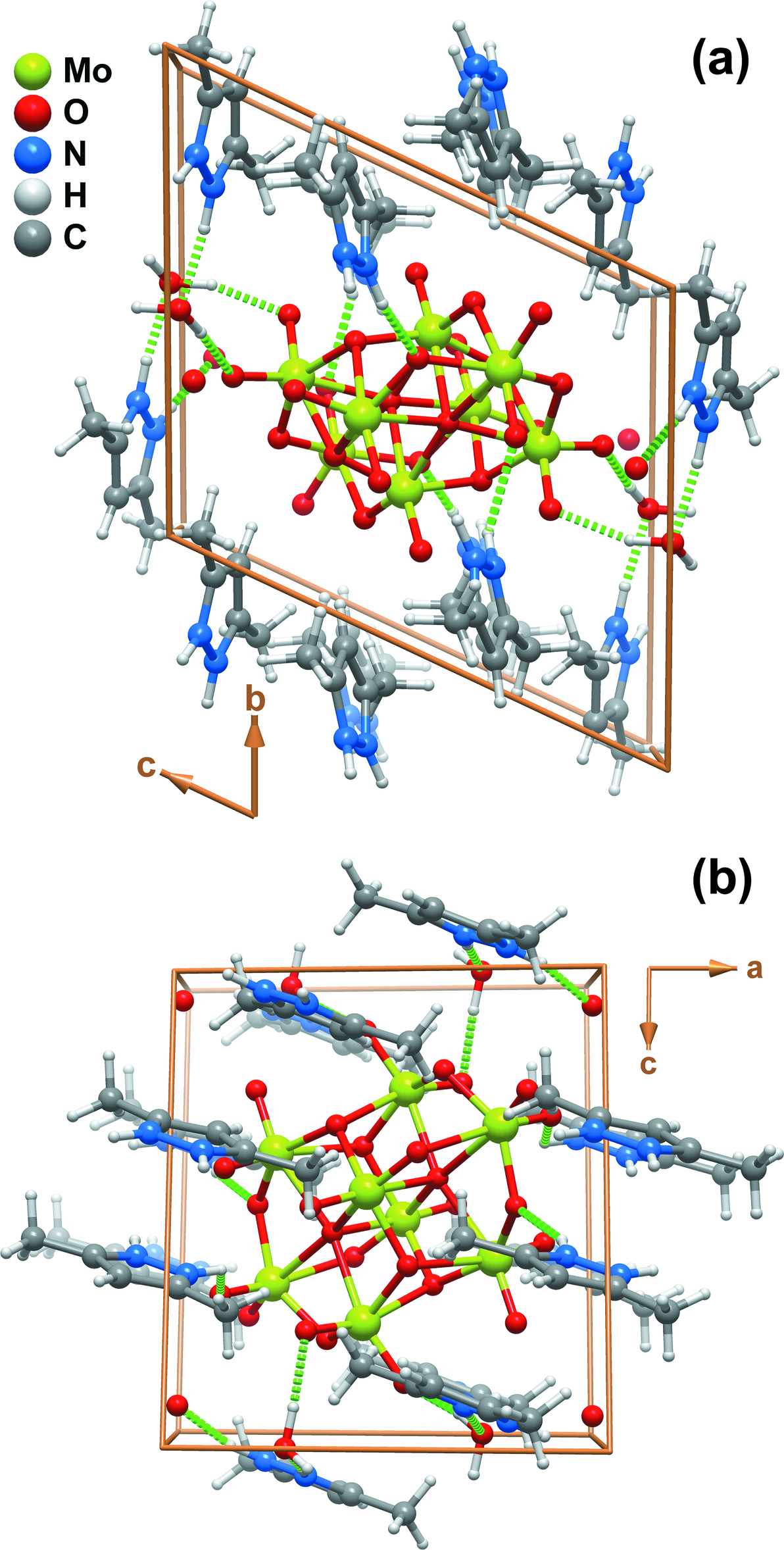

Supplement: Supplementary file 4 [file e-71-0m244-fig2.tif]

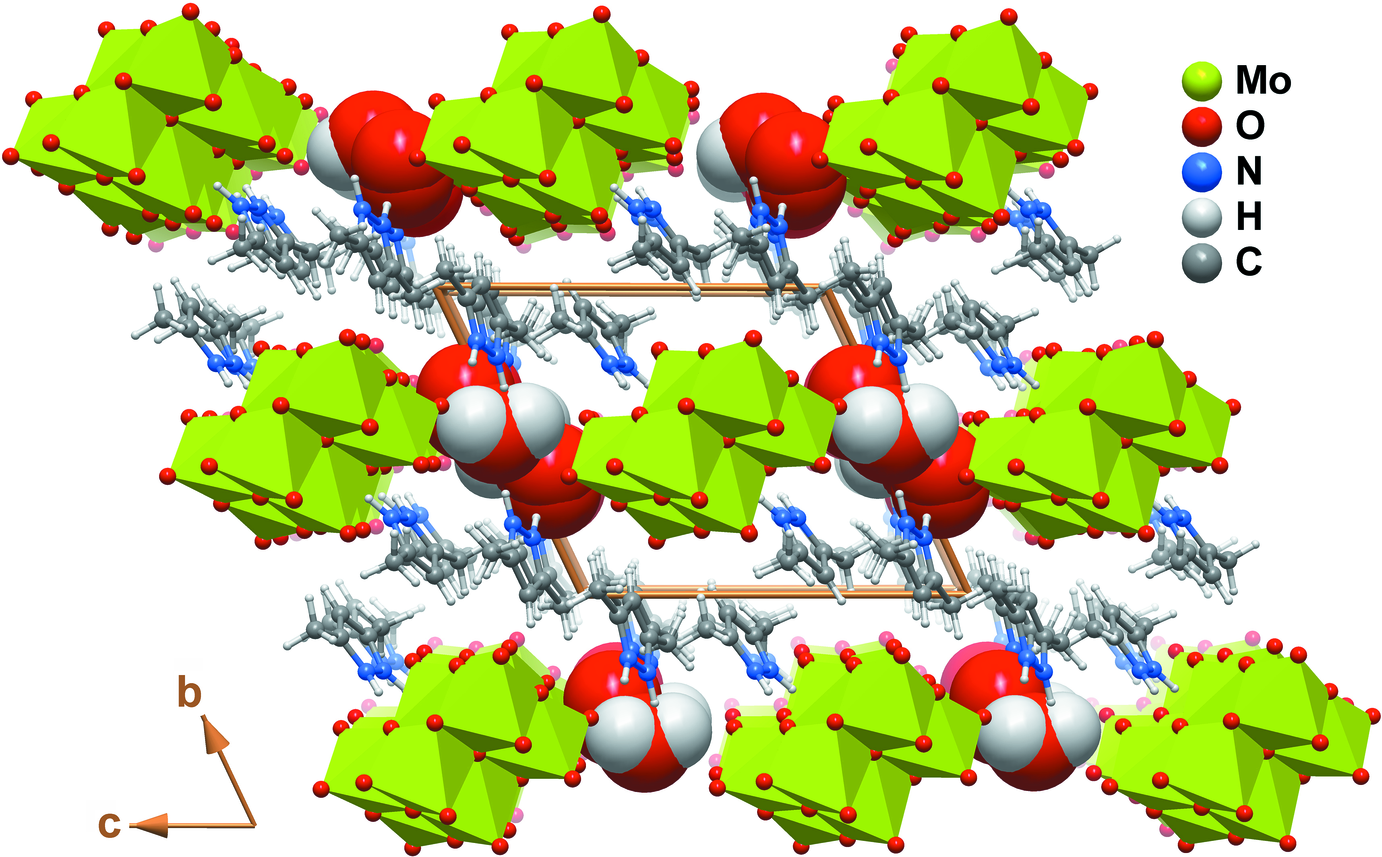

Supplement: Supplementary file 5 [file e-71-0m244-fig3.tif]
